# Inland Lakes Mapping for Monitoring Water Quality Using a Detail/Smoothing-Balanced Conditional Random Field Based on Landsat-8/Levels Data

**DOI:** 10.3390/s20051345

**Published:** 2020-02-29

**Authors:** Lifei Wei, Yu Zhang, Can Huang, Zhengxiang Wang, Qingbin Huang, Feng Yin, Yue Guo, Liqin Cao

**Affiliations:** 1 Faculty of Resources and Environmental Science, Hubei University, Wuhan 430062, China; weilifei2508@hubu.edu.cn (L.W.); 201711110811072@stu.hubu.edu.cn (C.H.); wangzx66@hubu.edu.cn (Z.W.); 201722110813024@stu.hubu.edu.cn (Y.G.); 2 Hubei Key Laboratory of Regional Development and Environmental Response, Hubei University, Wuhan 430062, China; 3 Key Laboratory of Urban Land Resources Monitoring and Simulation, MNR, Shenzhen 518034, China; 4 Shenzhen Cadastral Surveying and Mapping Office, Shenzhen 518000, China; chdd@pnr.sz.gov.cn; 5 Hubei Provincial Institute of Land and Resources, Wuhan 430070, China; 201911110811276@stu.hubu.edu.cn; 6 School of Printing and Packaging, Wuhan University, Wuhan 430079, China; clq@whu.edu.cn

**Keywords:** inland water, water quality levels, conditional random field, contextual information, Landsat 8 operational land imager (OLI)

## Abstract

The sustainable development of water resources is always emphasized in China, and a set of perfect standards for the division of inland water environment quality have been established to monitor water quality. However, most of the 24 indicators that determine the water quality level in the standards are non-optically active parameters. The weak optical characteristics make it difficult to find significant correlations between the single parameters and the remote sensing imagery. In addition, traditional on-site testing methods have been unable to meet the increasingly extensive water-quality monitoring requirements. Based on the above questions, it’s meaningful that the supervised classification process of a detail-preserving smoothing classifier based on conditional random field (CRF) and Landsat-8 data was proposed in the two study areas around Wuhan and Huangshi in Hubei Province. The random forest classifier was selected to model the association potential of the CRF. The results (the first study area: OA = 89.50%, Kappa = 0.841; the second study area: OA = 90.35%, Kappa = 0.868) showed that the water-quality monitoring based on CRF model is feasible, and this approach can provide a reference for water-quality mapping of inland lakes. In the future, it may only require a small amount of on-site sampling to achieve the identification of the water quality levels of inland lakes across a large area of China.

## 1. Introduction

Water is at the heart of sustainable development, and water resources play a vital role in meeting human productivity needs, economic development, environmental protection, and ecosystem services [1]. Globally, due to the geographical complexity of water supply and use, it is now difficult to assess whether sufficient freshwater resources are available for future needs [2]. However, what is certain is that a large proportion of the world’s population are currently suffering from “water stress” [3]. This stress is not only due to the shrinkage of freshwater resources, but it is also closely related to the large-scale pollution of surface water. Water pollution affects agricultural activities, human health, and the entire ecosystem [4].

Therefore, according to the environmental functions of surface waters and the goals of water resources protection, in China, the environmental protection departments have proposed a set of the standards of water quality rating. According to the Environmental Quality Standards for Surface Water (EQSSW, http://sthjt.hubei.gov.cn, 22 February 2020) [5], water quality in China can be divided into five levels, according to the water function, i.e., {I, II, III, IV, V} [6], which are determined by many parameters, including dissolved oxygen (DO), the potassium permanganate index, chemical oxygen demand (COD), ammonia nitrogen (NH_3_-N), total phosphorus (TP), and total nitrogen (TN), etc. The water quality level depends on the worst evaluation result of single parameters. When the water quality is lower than Class V, it is classified as Class VI. The Action Plan for the Prevention and Control of Water Pollution in China (State Council) pointed out that, by 2020, the overall proportion of the water bodies of seven basins (Yangtze River basin, Yellow River basin, Pearl River basin, Songhua River basin, Huaihe River basin, Haihe River basin, and Liao River basin) not meeting the Class III standard will be 70% or above. However, the Action Plan also requires that the proportion of the urban centralized drinking water sources reaching or exceeding Class III will be at least 95% (http://www.gov.cn/xinwen/2015-04/16/content_2847709.htm, 22 February 2020).

The sampling location and frequency of surface water quality monitoring in China are carried out in accordance with the requirements of the national technical specifications for surface water environmental monitoring. Monitoring sites on state-controlled water systems (rivers, lakes, and reservoirs) are sampled every two months, six times throughout the year, and the sampling points are densely distributed (HJ/T 91-2002, Technical Specifications Requirements for Monitoring of Surface Water and Waste Water, http://sthjt.hubei.gov.cn, 22 February 2020) [7,8]. The monitoring program includes 24 routine indicators. As a result, the surface water quality surveying consumes a lot of manpower and material resources every year, and the cycle is long. With the continuous advancement of remote sensing technology, more and more traditional investigation tasks can now be quickly solved by analyzing spaceborne images. For example, Landsat 8 OLI imagery was applied for land surface water mapping [9]. Landsat 8 and Landsat 7 were applied for regional measurements of chromophoric dissolved organic matter (CDOM) and water clarity in lakes [10]. Urbanski et al. [11] used Landsat 8 imagery to assess water quality based on regional scale. Furthermore, most of the images of the current multispectral series of Landsat satellites are available free of charge.

At present, surface water monitoring by means of remote sensing is mostly combined with ground sampling, and is extended to surface water monitoring by the quantitative retrieval of point source water quality parameters [12]. The spatial distribution of the water quality parameters is analyzed to investigate the pollution status of the water area [13,14,15,16]. However, most of the above retrieval targets are optically active substances such as chlorophyll-a [17], total suspended matter (TSM) [18], CDOM [19], etc. In the same environment, there will be more than one water quality variable that affects the remote sensing reflectivity. The optical properties of non-optically active parameters such as TP, TN, DO, etc., which determine water quality, are weak, and there is no significant correlation between remote sensing images and these parameters [13,20]. Furthermore, more than one parameter determines the level of inland water quality [21,22]. However, compared with the inversion of single water quality parameter, the classification of water quality levels based on remote sensing technology weakens the cumulative error of the individual water quality factors and strengthens the direct connection between water quality and remote sensing images. For the application research of traditional water quality monitoring, the classification process based on water quality levels is more suitable for the current scenario. As water quality level monitoring is a routine issue for environmental protection agencies, this process may be simplified in the future.

Artificial intelligence technology has developed rapidly, and supervised learning technology combining machine learning has become more and more mature. Because of generalization ability and good classification accuracy on most datasets, many scholars have combined random forest (RF) and remote sensing images for classification of water bodies [23]. These classifiers are based on pixel wise segmentation of images [24], and their classification results will be accompanied by salt and pepper noise [25]. However, the water area is often a connected whole, and the water quality attributes of the adjacent pixels are similar. Therefore, in the water quality classification of inland lakes, we can not only use spectral information of images, but also abundant spatial information. The conditional random field (CRF) method is a discriminant probability method that can effectively combine spatial information. The model is optimized on the basis of the Markov random field (MRF), and can consider contextual information in both label data and observation data [26]. For example, the authors of [27,28] used CRF model to integrate contextual information into remote sensing classification to improve classification accuracy and overcome salt-and-pepper classification noise. Since the many CRF-based models resulted in different degrees of smoothing [29], it is important to keep the details with the spatial contextual information.

In summary, although the amount of water on earth is huge (about 71% of the Earth’s surface is covered with water), the freshwater resources that can be directly used by humans are extremely rare (only 2.5% is fresh water, and 98.8% of this is in glaciers and groundwater) [30]. Therefore, surface freshwater resources, as one of the fundamental issues related to national economic development and ecological environmental protection, are receiving more and more attention from all countries [31]. Correspondingly, water pollution is one of the main causes of water shortages. Therefore, governments have become very concerned about the control and monitoring of water pollution. The classification of lake water quality based on satellite remote sensing will enable water pollution control to achieve rapid positioning and precise treatment. In this paper, a classification method for water quality levels based on CRF and RF model is proposed. By the use of Landsat 8 OLI imagery, the lakes of two study areas in Hubei Province, China, were classified, and the experimental results of the based-pixel RF, decision tree (DT), and deep neural network (DNN) models were compared.

## 2. Materials and Methods 

### 2.1. Study Areas

**Figure 1:**
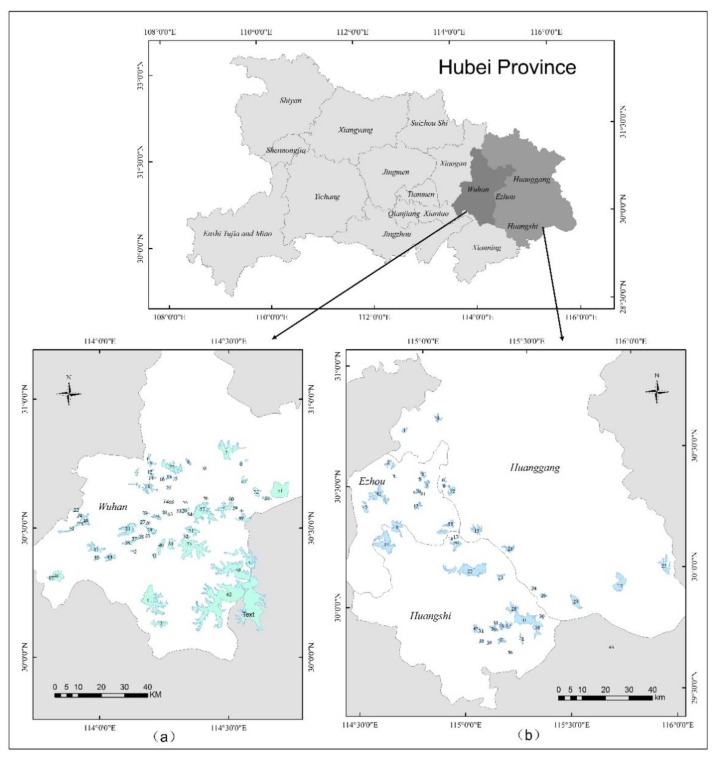
Study areas: (**a**) the Wuhan study area; (**b**) the Huangshi study area.

In this study, the water system of Wuhan and its surrounding areas was selected as the first study area (Wuhan dataset). The second study area was around Huangshi and along the Yangtze River system (Huangshi dataset). Located in central China, Wuhan is situated at the intersection of the Yangtze River and its tributaries, and is China’s largest economic, cultural, and educational center. The city covers an area of 8549 square kilometers, 25% of which is covered by lakes, shallow waters, canals, and rivers. The surface water resources in the area are therefore very rich [32,33]. In this study, an area containing 76 lakes was selected as the research area in Wuhan, as shown in Figure 1a. 

The lakes selected for the second experimental area are located on both sides of the Yangtze River, near the city of Huangshi, and total 49 in number (Figure 1b). According to the statistics of the Changjiang River Scientific Research Institute of the Changjiang Water Resources Commission, the discharge of wastewater in the Yangtze River Basin increased from 19.7 billion tons in 1998 to 35.3 billion tons in 2016. Each year, about 33 billion tons of agricultural wastewater containing high levels of nutrients flows into the Yangtze River system, and the water pollution problem is serious [34]. Xu Kuangdi, said that, in the construction of ecological civilization, the urban agglomeration in the middle reaches of the Yangtze River should take water resources protection as the core, and an ecological corridor with clear water, green land, and blue sky should be built in the cities [35].

### 2.2. Satellite Data and Vector Data 

NASA successfully launched the Landsat 8 satellite on 11 February 2013, with the OLI sensor onboard, which collects data from nine spectral bands. Apart from the 8th panchromatic band (15 m), the remaining bands have a spatial resolution of 30 m. The first seven commonly used bands were selected for the experimentation, i.e., coastal, blue, green, red, NIR, SWIR1, and SWIR2 [36,37]. The Landsat 8 OLI images were obtained from the Geospatial Data Cloud site (http://www.gscloud.cn/search, 22 February 2020).

The data identifier of the image selected for the first study area of Wuhan is LC81230392018098LGN00. The image was acquired on April 8, 2018, when the amount of cloud was 8.94% and the lakes were not covered by cloud. The data identifier of the image selected for the second study area of Huangshi is LC81220392017120LGN00. This image was acquired on 30 April 2017, with 0.06% cloud cover. The lake vector maps within the two research areas were also obtained from the Geospatial Data Cloud site, for the water extraction of the Landsat 8 images.

### 2.3. Surface Water Environment Quality Levels

The water quality data of the first study area (Wuhan) from May 2018 were obtained from the Wuhan Ecology and Environment Bureau (http://hbj.wuhan.gov.cn/hbHjjc/index.jhtml, 3 January 2020). The water quality data of the second study area (Huangshi) were released by the provincial environmental monitoring center station of the Department of Ecology and Environment of Hubei province in 2017 (http://sthjt.hubei.gov.cn, 22 February 2020). The water quality data is the result of in-site sampling in a certain period. In order to ensure the validity of the experimental data, the acquisition time of the selected Landsat 8 OLI images should be similar to the collection time of the water quality data. Several representative lakes in the first and second study areas were selected in Table 1 and Table 2 respectively. The parameters of over-standard in the assessment of the water quality were pointed out in the table, including TP, COD, biochemical oxygen demand (BOD), Permanganate index (COD_Mn_) and NH3-N. Surface water is divided into five level (Table 3) according to the function. The standard values for the respective functional class are executed. The Class I water belongs to the source water, which belongs to the national natural protection zones. The water quality in the two table represents the current level of water quality assessed for the lakes. The value represents the superstandard multiple, and the datum line correspond to the function level of the lakes.

**Figure 2:**
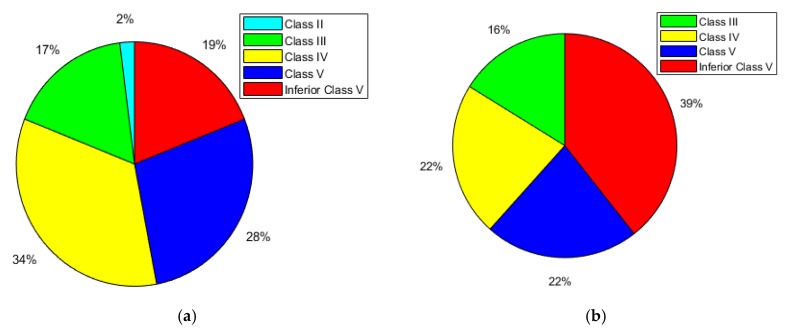
Percentage of lakes in each water quality level: (**a**) Wuhan; (**b**) Huangshi.

The pie charts in Figure 2 show the percentage of lakes in each water quality level, for both study areas. In Wuhan and its surrounding areas, we recorded the water quality levels of 64 lakes, as shown in Figure 2a. In this area, there are no Class I lakes (the highest water quality). The Class II lakes number only one, namely, Niushan Lake. The Class III lakes account for 17%, and the Class IV, Class V, and Class VI lakes account for 34%, 28%, and 19% respectively. Therefore, only 19% of the lakes meet or exceed the Class III water quality level, which is the standard for centralized drinking water. In the second study area of Huangshi, along the lower reaches of the Yangtze River, 49 lakes were selected on both sides of the Yangtze River for the statistical analysis, as shown in Figure 2b. In this area, there are no Class I or Class II waters. Class III lakes account for about 16%, Class IV and Class V lakes both account for about 22%, and the Class VI lakes account for about 39%. Therefore, only 16% of the lakes are equal to or better than the Class III water quality level.

### 2.4. Methods

#### 2.4.1. The Improved Conditional Random Field (CRF) Model and Other Models

The CRF is a kind of probability model, which has been widely used in image segmentation, stereoscopic vision and activity analysis because of its ability to combine spatial information [29]. In this paper, a method of water-quality classification based on the detail-preserving smoothing CRF was proposed, which used the probability of each class obtained by the RF classifier to define as the unary potential of the CRF, and defined the linear combination of the spatial smoothing term and the local class label cost term as the pairwise potential, so as to achieve the classification effect of combining spatial contextual information and retaining detailed information at the same time.

The CRF model have been developed with a unified probability framework to simulate local neighborhood interactions between random variables, where the posterior probability is expressed as a Gibbs distribution directly [38]:

(1) $$P(x|y)=\frac{1}{Z(y)}\exp\left\{-\sum_{c\in C}\psi_{c}(x_{c},y)\right\}$$

where *y* is the observation data of the input image, that is, the pixel-by-pixel spectral vector; *x* represents the class labels; *Z(y)* is the partition function; $\psi_{c}(x_{c},y)$ is the potential function, which models the spatial interaction of random variables locally based on the neighborhood system and clique *c* in the image; and *C* represents a fully connected subgraph. In this paper, 8-neighborhood model was applied in pairwise CRF framework.

Assuming an observation filed $y=\{y_{1},y_{2},\cdots ,y_{N}\}$, which *N* is the total number of pixels, and a labeling field $x=\{x_{1},x_{2},\cdots ,x_{N}\}$. According to the posterior distribution of the label *x*, given the observation *y*, the corresponding Gibbs energy is shown in Equation (2):

(2) $$E(x|y)=-\log P\left(x|y\right)-\log Z\left(y\right)=\sum_{c\in C}\psi c\left(xc,y\right)$$

In order to find the label image x which maximizes the posterior probability P(x|y), based on the Bayesian maximum posterior rule (MAP), the MAP label $X_{MAP}$ of the random field is given:

(3) $$X_{MAP}=\underset{x}{argmaxP\left(x|y\right)}=\underset{x}{\arg\min E\left(x|y\right)}$$

When the posterior probability P(x|y) is maximum, the energy function E(x|y) is minimum. In Equation (3), $E(x|y)=\sum_{i}\phi_{1}(x_{i},y)+\lambda \cdot \sum_{i<j}\phi_{2}(x_{i},x_{j},y)$, $\phi_{1}$ is the unary potential function, which represents the segmentation result under the premise of independent consideration of each pixel; $\phi_{2}$ is the pairwise potential function, which represents the influence of the relationship between pixels on segmentation. The nonnegative λ is the tuning parameter that represents the proportion of the pairwise potential. The larger λ, the more obvious the smoothing effect.

The unary potential function models the relationship between class label and pixel spectral data. The probability estimation of each pixel is calculated by discriminant classifier, and the feature vectors are given. It plays a leading role in the process of classification, and is generally the posterior probability of a supervised classifier. The unary potential function is defined as:

(4) $$\phi_{i}\left(x_{i},y\right)=-\ln\left\{P\left[x_{i}=l_{k}|f_{i}\left(y\right)\right]\right\}$$

where $f_{i}\left(y\right)$ represents the feature vector at the position *i*, which comes from the spectral dimension mapping of a pixel in an image. $P[x_{i}=l_{k}|f_{i}(y)]$ is the probability of class label $l_{k}$ taken by the pixel i based on the feature vector. Because the RF algorithm is stable, and the classification effect is good without parameter adjustment, the RF classifier was selected as the unary potential.

Based on the probability distribution results of the unary potential, the pairwise potential function models the label class relationship of the pixels in the neighborhood. The similarity between pairs of pixels is measured by the local features of the image, which affects the label class between pixels in the neighborhood, and reflects the interaction of points. In order to minimize the Gibbs energy of the corresponding model, if the feature difference between pixels is large, the pairwise potential function value should be small, that is, the labeling results should be accepted; If the feature difference between pixels is small, the pairwise potential function value should be large, and the labeling results should be modify by the model. The expression of the pairwise potential function is:

(5) $$\psi_{ij}\left(x_{i},x_{j},y\right)=\{\begin{array}{cc} 0 & if\;x_{i}=x_{j}\\ g_{ij}\left(y\right)+\theta *\Theta L\left(x_{i},x_{j}|y\right) & otherwise \end{array}$$

where $g_{ij}\left(y\right)$ represents a smooth term related to data y, $g_{ij}\left(y\right)=dist{\left(i,j\right)}^{-1}\exp\left(-\beta {\left\|y_{i}-y_{j}\right\|}^{2}\right)$. dist(i,j) is the Euclidean distance, and y is the spectral vector. $\Theta_{L}\left(x_{i},x_{j}|y\right)$ represents the cost between labels $x_{i}$ and $x_{j}$ in the neighborhood. The parameter θ is applied to control the degree of the label cost term in pairwise potential function. The range of parameter θ is usually [0–4]. The local class label cost term $\Theta_{L}\left(x_{i},x_{j}|y\right)$ is defined as:

(6) $$\Theta L\left(x_{i},x_{j}|y\right)=\frac{\min\left\{P\left[x_{i}|f_{i}\left(y\right)\right],P\left[x_{j}|f_{j}\left(y\right)\right]\right\}}{\max\left\{P\left[x_{i}|f_{i}\left(y\right)\right],P\left[x_{j}|f_{j}\left(y\right)\right]\right\}}$$

where $P\left[x_{i}|f_{i}\left(y\right)\right]$ is the label probability given by the RF classifier; $f_{i}\left(y\right)$ represents the spectral feature vector at the position *i*; and $x_{i}$ is the class label. $\Theta_{L}\left(x_{i},x_{j}|y\right)$ will affect the label estimation of the current pixel according to the probability distribution of adjacent pixels, so the model can smooth the classification results when considering the spatial contextual information.

As mentioned earlier, the local class label cost term is expressed as a probability estimate of the spatial distribution of category labels. Thus, the final classification accuracy depends on the accuracy of the probability estimate, which is obtained from majority voting of the original RF classification map. In order to effectively remove the salt-and-pepper classification noise, the label property of adjacent cells should be taken into account. Therefore, the maximum of all the class labels for each pixel will be the probability estimate of the segmentation result.

In summary, aiming at the classification of water quality in China’s water quality assessment system, a supervised classification method CRF combined spectral information with spatial contextual information was proposed in this paper. It takes the probability distribution of the RF classifier as the unary potential, and defines the linear combination of spatial smooth term and local label cost term as pairwise potential. The model can predict the label class of current pixel with reference to the water quality level of adjacent pixel. In addition, three pixel-based classifiers were added to the experiment.

Three other models were discussed in this paper, namely the based-pixel RF, DT and DNN. For the image classification problem, the RF is not the best-performing algorithm. However, due to its simplicity, ease of implementation, strong generalization ability, and good performance on many datasets, it has been widely used in academic research and industrial applications [39,40]. The RF is an algorithm that integrates multiple trees through the idea of ensemble learning. Its basic unit is the decision tree, so N trees have N classification results. RF integrates N voting results, and the class with the most votes is the final output. Recently, although the DT model is no longer a mainstream classifier for use alone, it is widely used as a base learner in more complex algorithms, because of its fast speed, high accuracy, and ease of understanding [41,42]. DT classification represents the process of classifying instances based on features. Based on the if-then rule, its classification speed is fast and it is a commonly used classifier. Since the number of samples is not uniform, the DT model automatically adjusts the weight based on the number of samples. LeCun et al. [43] published an article on deep learning in *Nature* in 2015, expressing the importance of the model to human society. The DNN is a pixel-based supervised learning model and the basis of other deep learning models. The ability of the neural networks to express models is dependent on the optimization algorithms. The optimizer selection will be described further later. The training process of a DNN consists of two parts: the forward propagation of the signal and the reverse propagation of the error. The back-propagation algorithm can optimize the weight and bias of the neural network according to the defined loss function, so that the loss value of the model reaches a smaller value. In this study, the algorithms were implemented in Python and TensorFlow.

#### 2.4.2. Evaluation Indicators

The overall accuracy (OA) and Kappa coefficients (Kappa) were applied to evaluate the results of the model predictions in the experiments, and were calculated based on a confusion matrix. The OA represents the percentage of all the samples with correct predictions divided by all the samples which take part in the classification. That is, the number of oblique diagonal samples of the confusion matrix divided by the total number of samples. Because there was no significant priority between the different water quality levels in these experiments, and the classification result of a certain level has no decisive influence on the evaluation of the model, the OA can express the classification accuracy most intuitively, so it was applied in these experiments. Kappa is used for consistency testing and is often used for multi-classification problems [44,45]. The formula for Kappa is:

(7) $$k=\frac{p_{oa}-p_{e}}{1-p_{e}}$$

(8) $$p_{e}=\frac{a_{1}\cdot b_{1}+a_{2}\cdot b_{2}+\cdots +a_{c}\cdot b_{c}}{n\cdot n}$$

where $p_{oa}$ represents the overall classification accuracy OA, $a_{i}$ is the number of ground-truth samples of the *i*-th class, $b_{i}$ is the number of samples predicted by the *i*-th class, and *n* represents the total number of samples. Kappa takes a range of [0, 1] in practical applications. The higher the coefficient value, the higher the accuracy of the model prediction results.

The ultimate purpose of this study is to use only a few samples to predict the water quality levels of the lakes. Therefore, it is necessary to count the number of each class label within the vector range of each lake. The classification result of water quality is determined by the maximum percentage of the labels. When the lake is classified as a unit, the results are still evaluated by OA.

## 3. Experiments and Analysis

### 3.1. Data Description

**Figure 3:**
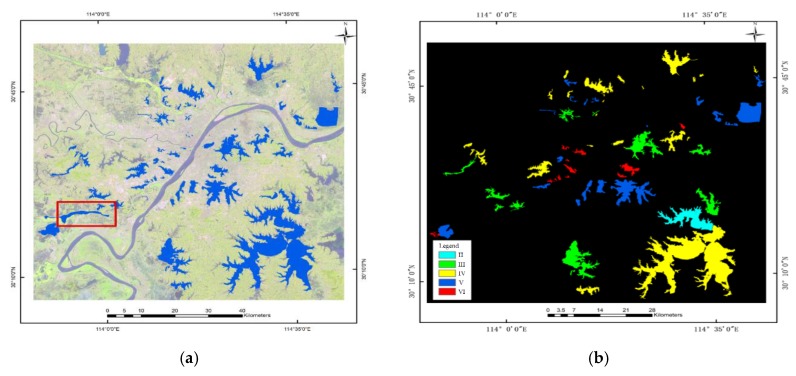

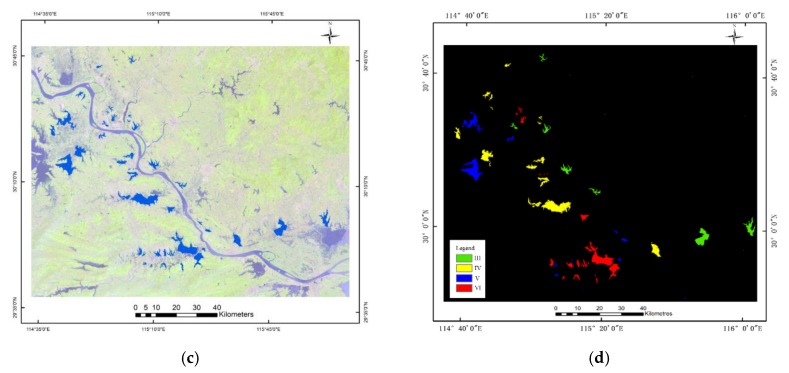
The datasets of the two study areas. (**a**) False-color composite and water vector data for the Wuhan study area. (**b**) ground-truth map in the Wuhan study area. (**c**) False-color composite and water vector data for the Huangshi study area. (**d**) ground-truth map in the Huangshi study area.

According to the vector maps, most of the lakes were divided into water quality levels, except for some lakes where the range of the water quality levels was uncertain. The experimental datasets were subjected to water vector masking and water extraction. The statistical results are shown in Table 4. The colors in Table 4 correspond to the water quality level distributions in Figure 3, and the last column is the total number of samples in the datasets of the two study areas. It can be observed that the number of each label class is unevenly distributed. In the second study area, the Class VI lakes are mainly concentrated in densely populated cities on both sides of the Yangtze River. These lakes are small and their circulation is poor, so they are easily polluted. In the second study area, the numbers of samples for each level were relatively uniform (between 18% and 34%). In the experiment, the datasets were produced as the form [features, samples], where the features refer to the seven bands of the images, and the sample represents a single pixel. Because the number of image bands is small, we used all bands for model training without too much information redundancy. In addition, because RF, DT, and DNN, including CRF’s unary potential, are all based on pixels, the data sets produced are also based on pixels. The training set: test set = 1:9, where the training set was applied to learn the parameters of the model, and the test set was used to test the classification effect of the model.

The image sizes for the first and second study areas were 2873 × 3037 pixels and 4121 × 4784 pixels, respectively. Figure 3a,c show the false-color images and the water vector data (blue) from the Geospatial Data Cloud. Figure 3b,d are the distribution maps of the lakes after water extraction, that is, corresponding ground-truth maps. In order to ensure the effectiveness of the extracted lake range, a masking process was performed using the water body vector (blue) before water extraction. Then the ground-truth maps were used as a mask file to extract the range of the study area on the Landsat images. Finally, we applied the masked images to generate the above mentioned datasets through MATLAB.

### 3.2. Experiment 1: The Wuhan Dataset 

The first experiment was conducted for the Wuhan study area and its surrounding water systems. To evaluate CRF performance, we compared its classification performance with that of common machine-learning models DT, DNN and RF. In total, 100 trees were set in the experiment for RF. The minimum number of samples required to split an internal node was 10. Since only the seven bands of the Landsat 8 OLI image were used, we did not need to set the maximum number of features for the DT and RF. For the optimizer of the DNN (Table 5), we considered stochastic gradient descent (SGD), momentum, and the adaptive optimizer of root mean square prop (RMSProp), but their effects were slightly worse than that of the adaptive moment (Adam) optimizer. The models trained by the Adam optimizer were stable and highly accurate. The neural network structure was manually adjusted, and the four layers of hidden layers were determined. The number of neurons in each layer was 2^8^. The predictive accuracy of the model trained by this structure was found to be the highest. The learning rate was set to 0.01. Slightly larger values caused the loss value to fluctuate significantly. The number of iterations was 2000.

**Figure 4:**
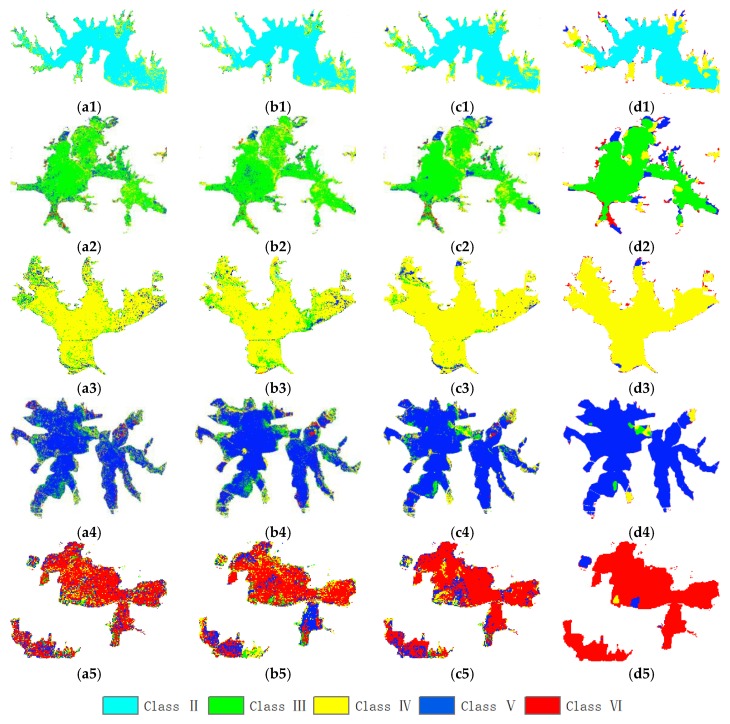
The classification result of the water quality levels of the lakes for the Wuhan dataset: (**a1**–**a5**) DT; (**b1**–**b5**) DNN; (**c1**–**c5**) RF; (**d1**–**d5**) RF-CRF; (1) Class II Niushan Lake; (2) Class III East Lake; (3) Class IV Wu Lake; (4) Class V Tangxun Lake; (5) Class VI South Lake and Yezhi Lake.

The adjustable parameters in the CRF model include the weight of the pairwise potential λ. Through the experiments, the effect of the parameter λ on the experimental results is very obvious, which was determined to be 0.8 in the two study areas. In addition, the scope of the pairwise potential is 8 adjacent pixels. Since a segmentation method similar to object-oriented processing is used, which was mentioned above section, the salt and pepper phenomenon has been alleviated a lot. In Figure 4, (c1–c5) is the probability estimation of the pixel-oriented RF obtained from the unary potential, and (d1–d5) are the output maps of the segmented result of the pairwise potential. It is observed that the classification accuracy for the water-quality levels of a single lake is greatly improved.

Table 6 lists the classification accuracies (OAs) of the different classifiers for each water quality level. For the Class VI water, the classification result of the RF-CRF classifier is much better than other classifiers. And whether it is OA (89.5%) or kappa (0.841), the accuracy of this classifier is also the highest. A representative lake was selected for each type of label in Figure 4, including Niushan Lake (Class II), East Lake (Class III), Wu Lake (Class IV), Tangxun Lake (Class V), South Lake and Yezhi Lake (Class VI). Since the DT, DNN, and RF models are classified on the pixels, a lot of salt-and-pepper noise can be seen from the figure. Especially for South Lake, although the RF classification result is slightly worse than the CRF, the misclassification scene still stands out. However, because the CRF model has used spatial contextual information, and the label information of adjacent pixel can also affect the classification results of the current pixel, it can be seen that the smoothing effect of the classification maps of the lake is very obvious. This will be of great help to further judge the water quality level of the lakes based on the number of class labels.

**Figure 5:**
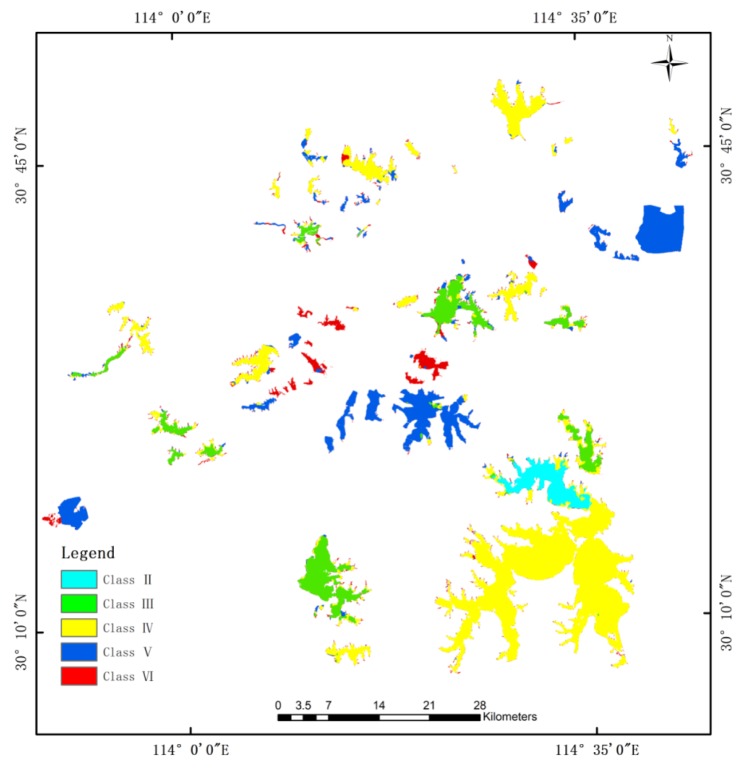
The classification result of the RF-CRF model for the Wuhan dataset.

The spatial distribution of the water quality level based on the RF-CRF in the first study area is shown in Figure 5. According to the Table 6, the OA of the Class VI water is 95.34%. From the red area in the Figure 3, the Class VI lakes are mainly distributed on both sides of the Yangtze River and in the densely populated urban areas. The several Class VI lakes can be predicted accurately, and the water quality in the whole range of lakes is clearly presented. However, the classification of small lakes will still cause serious misjudgments. Due to the influence of adjacent pixels, there are more misclassifications in the classification of lake edge regions. This has a very large impact on the prediction results of the finely divided patches.

According to the classification result maps of the water quality levels, within the vector range of each lake, the number and percentage of the pixels of each water quality level were counted. The class with the largest proportion was then considered as the water quality level of the lake, as shown in Table 7. As shown in the table, excluding individual lakes whose original water vectors do not match the image, there are 64 lakes in the first study area for experiments. The bold figures are the number of lakes correctly predicted. There is only one lake in the Class II lakes, which was correctly predicted. There are 11 lakes with a water quality of Class III, which were predicted 81.8% correctly. In total, 21 Class IV lakes were accurately identified. In addition, one lake was classified as Class V. There are 20 lakes in Class V, except for two lakes that was not extracted, and the remaining 18 are correctly identified. And Class VI lakes were fully predictive and accurate. In summary, within the study area, the number of correctly predicted lakes in the lakes used for the experiment reached 61, and the accuracy rate was 95.31%. Most of the lakes that were judged to be the wrong class were relatively small. 

### 3.3. Experiment 2: The Huangshi Dataset

The second experiment was conducted for the city of Huangshi and the surrounding water systems in the lower reaches of the Yangtze River. The classification accuracies for the four levels are listed in Table 8. For RF, the number of trees (100) and the minimum number of samples required to split an internal node (10) were adjusted using the same adjustment strategy as that used in the first study area. The strategy of superparametric adjustment of the DNN model was the same as for the first study area. The structure of the neural network was manually adjusted. The number of hidden layers is 4, and the number of neurons in each layer is 2^8^. The Adam optimizer was selected, with a learning rate of 0.01, an iteration number of 2000, and 10% of the neurons were randomly deleted. The RF-CRF achieved the best classification performance (90.35% of OA, 0.868 of Kappa), higher than the DT-based, DNN-based and the RF-based methods. Its Kappa is 0.047 higher than that of RF. The highest prediction accuracy for the samples of the four levels is 99.06%, and there is no obvious class with a high misclassification ratio. 

**Figure 6:**
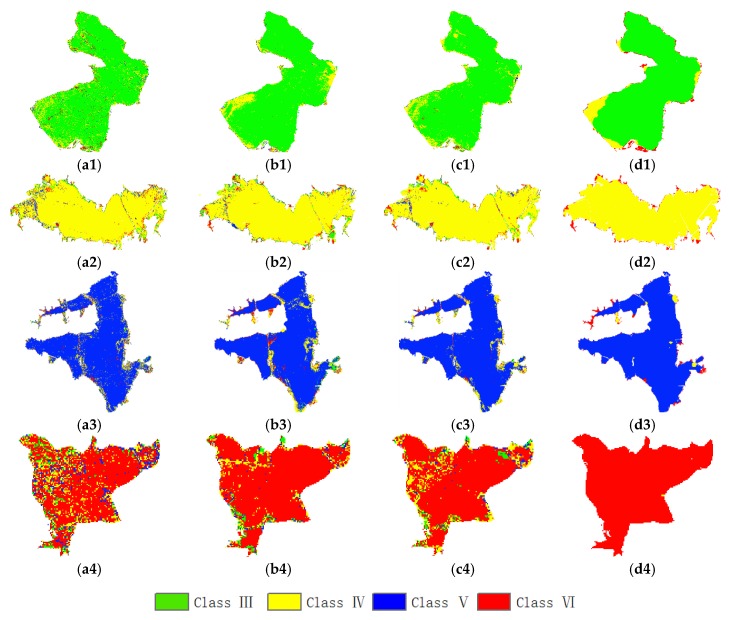
The classification result maps of the water quality levels for the Huangshi dataset: (**a1**–**a4**) DT; (**b1**–**b4**) DNN; (**c1**–**c4**) RF; (**d1**–**d4**) RF-CRF; (1) Class III Taibai Lake; (2) Class IV Daye Lake; (3) Class V Baoan Lake; (4) Class VI Haikou Lake.

Based on the trained four models, the water quality for the second study area was predicted. A representative classification result is selected from the lakes of each water quality level for display in Figure 6. From the perspective of the mapping effect, the CRF still performs best, followed by the RF. The mapping results are consistent with the calculated accuracy of the classifier. Haikou Lake is located in Huangshi City, which is directly connected to the Yangtze River. The field test results of the water quality showed that the phosphorus exceeded the standard, and the performance was Class VI. Figure 6(c4) is the RF classification result of Haikou Lake. Although the RF classification accuracy is not much different from the RF-CRF, there is still a certain phenomenon of salt and pepper. It is known during the manual interpretation that the class labels of all the pixels in the same lake are the same, but the pixel-based classification method ignores this important information. And the RF-CRF used the correlation information of adjacent pixels in the water images, so that all the pixels of Haikou Lake were completely predicted accurately. In addition, the performance at Baoan Lake and Daye Lake was also excellent.

**Figure 7:**
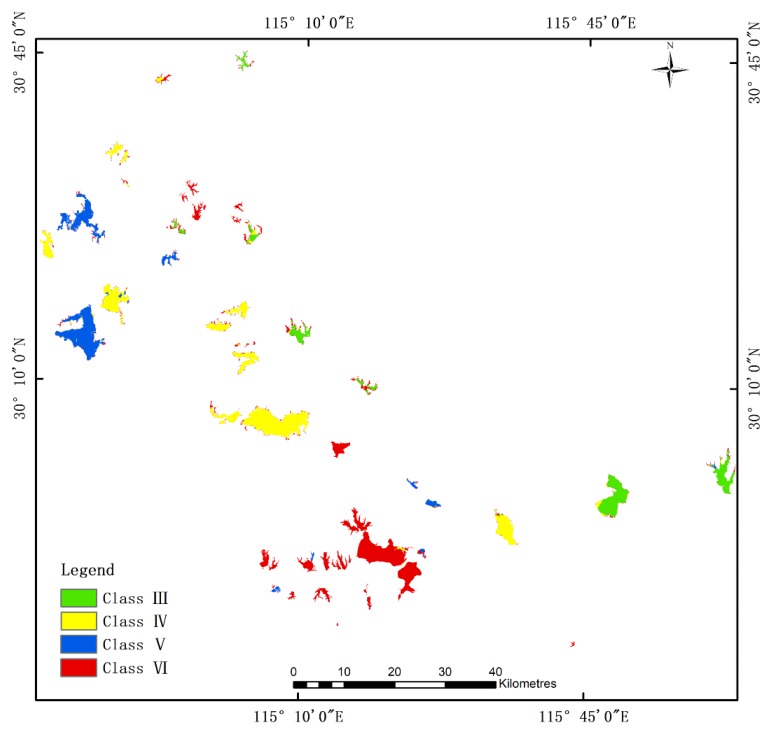
The classification result of the RF-CRF model for the Huangshi dataset.

The spatial distribution of the water quality level based on the RF-CRF in the second study area is shown in Figure 7. The Class VI lakes (red) are concentrated on both sides of the Yangtze River, and most of them are located in Huangshi City. Combined with Table 8, the accuracy of its prediction is close to 100%, which is much higher than DT (80.62%), DNN (75.97%), and RF (84.37%) models. Going to the upper reaches of the Yangtze River in the Ezhou, the water quality has improved significantly, and most of them are Class IV water. However, the situation at the edge of the lake can still be seriously mispredicted. Especially small lakes, it is difficult to determine the water quality of the lake as a whole. In future research, it will be necessary to adopt a satellite with a high resolution.

According to the classification result maps of the water quality levels for the Huangshi dataset, the class with the largest proportion is regarded as the water quality level of the lake, as shown in Table 9. The number of Class III and Class VI lakes is 8 and 19 respectively, and the accuracy all reaches 100%. The classification accuracy of Class IV and Class V lakes is 63.6%. It is found through statistical observation that most of the misclassified lakes are extremely small lakes, and the noise phenomenon is obvious. Because the CRF model classifies based on contextual information, the finer patches will reduce the classification effect. In addition, since the unary potential of CRF is pixel-based RF model, the bottom reflection of the lake will directly affect the probability distribution of class labels, and then affect the final classification results of the CRF model. Therefore, application of the RF-CRF to remotely sense water-quality levels needs to be further strengthened, and it is necessary to improve the spatial resolution of images in future experiments.

## 4. Conclusions

In this paper, in view of the China’s water quality assessment system, we discussed the possibility of using Landsat 8 imagery and machine learning methods to assess the water quality of inland lakes on a large scale. Due to the spatial continuity of the lake, the CRF classifier based on the probability map is suitable for the current scene. The RF was applied as the unary potential of the CRF model. To evaluate CRF performance, three other commonly used pixel-based classifiers were selected for comparison. The classification accuracy of the RF-CRF model was found to be the highest, with the Kappa in the two study areas being 0.841 and 0.868, respectively. We also investigated the prediction effect of the RF-CRF model on the whole images. 

The ultimate goal of this paper was to explore the possibility of using satellite imagery and machine learning algorithms to rapidly evaluate lake water quality levels. By training a small number of samples, we can predict the water quality of a large range of lakes and basically determine the water quality level of each lake. This approach not only has great benefits with regard to time cost, but is also represents a breakthrough in the application of remote sensing technology in water quality monitoring. And this methodology could be used by authorities on the water quality monitoring program in China. Through continuous exploration of the possibility of the CRF applied for water quality classification, further improving the accuracy of classification, training a more stable model with strong mobility, the monitoring team will be able to regularly assess the water quality of inland water using the satellite imagery rapidly.

In the future, satellites with a high spatial resolution and short revisit period could be utilized in water quality monitoring, and it will be possible to explore emergency response strategies via water quality remote sensing. Although the CRF considered in this paper were based on the characteristics of the spectral dimension and spatial dimension for classification. But some more valuable spatial features (textures, edges, etc.) are not used. Therefore, in future experiments, extraction of spatial features will be considered, and spatial-spectral fusion technology will be applied to further improve classification accuracy. 

## Figures and Tables

**Table 1:** Water quality data of the major lakes in Wuhan.

Lakes	Water Quality	Assessment (Superstandard Multiple)				
		TP	COD	BOD	COD_Mn_	NH3−*N*
Lu Lake	III	0.72	0.15	—	—	—
Houguan Lake	IV	—	0.30	0.15	—	—
Tangxun Lake	V	2.42	0.16	0.02	—	0.84
South Lake	VI	0.87	—	—	—	0.41

**Table 2:** Water quality data of the major lakes in Huangshi.

Lakes	Water Quality	Assessment (Superstandard Multiple)				
		TP	COD	BOD	COD_Mn_	NH3−*N*
Wushan Lake	IV	0.6	—	0.1	—	—
Baoan Lake	V	1.2	—	—	—	—
Qinggang Lake	VI	6.5	0.6	1.0	0.4	—

**Table 3:** Main parameter standard of water quality classification.

Parameters	Water Quality Class (mg/L)				
	I	II	III	IV	V
TP (Lake) ≤	0.01	0.025	0.05	0.1	0.2
COD ≤	15	15	20	30	40
BOD ≤	3	3	4	6	10
COD_Mn_ ≤	2	4	6	10	15
NH3−*N* ≤	0.15	0.5	1.0	1.5	2.0

**Table 4:** Water quality levels and numbers of samples in the Wuhan and Huangshi datasets.

Study Area	Class			Sample	
	No.	Color	Water Quality Level		Numbers
Wuhan	1	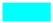	Class II		52,781
	2	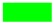	Class III		142,509
	3	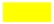	Class IV		370,184
	4	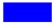	Class V		146,031
	5	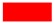	Class VI		25,115
		Total		736,619	
Huangshi	1	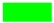	Class III		73,386
	2	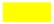	Class IV		137,606
	3	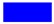	Class V		85,689
	4	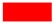	Class VI		105,462
		Total		402,143	

**Table 5:** The characteristic of the optimizers of DNN.

Optimizers	Characteristic
SGD	Parameter update speed is fast, but the vibration range is large
Momentum	Restrain vibrate, but poor adaptability
RMSProp	Solve the problem of sharp drop in learning rate and reduce manual adjustment of learning rate
Adam	Calculate the adaptive learning rate of each parameter with good adaptability

**Table 6:** Comparison of the different classification accuracy results (%) for the Wuhan dataset.

Level	DT	DNN	RF	RF-CRF
Class II	80.30	81.89	77.74	75.93
Class III	70.46	75.38	74.02	73.31
Class IV	85.33	88.27	93.22	95.21
Class V	77.36	78.50	83.55	94.70
Class VI	59.81	55.75	67.76	95.34
OA (%)	79.64	82.27	85.61	89.50
Kappa	0.693	0.731	0.778	0.841

**Table 7:** The statistics of the prediction results for the water quality levels in each lake for the Wuhan dataset.

No.	Water Quality Levels	Lakes (Number)	OA	Prediction				
				1	2	3	4	5
1	Class II	1	100%	**1**	0	0	0	0
2	Class III	11	81.8%	0	**9**	0	1	1
3	Class IV	22	95.5%	0	0	**21**	1	0
4	Class V	18	100%	0	0	0	**18**	0
5	Class VI	12	100%	0	0	0	0	**12**

**Table 8:** Comparison of the different classification accuracy results (%) for the Huangshi dataset.

Levels	DT	DNN	RF	RF-CRF
Class III	78.30	78.66	82.38	79.13
Class IV	82.03	84.96	88.22	90.00
Class V	80.62	75.97	84.37	89.79
Class VI	83.50	87.90	90.19	99.06
OA (%)	81.44	82.66	86.85	90.35
Kappa	0.747	0.763	0.821	0.868

**Table 9:** The statistics of the prediction results for the water quality levels in each lake for the Huangshi dataset.

No.	Water Quality Levels	Lakes (Number)	OA	Prediction			
				1	2	3	4
1	Class III	8	100%	**8**	0	0	0
2	Class IV	11	63.6%	0	**7**	0	4
3	Class V	11	63.6%	0	0	**7**	4
4	Class VI	19	100%	0	0	0	**19**
